# Plasma Leptin Levels and Risk of Incident Cancer: Results from the Dallas Heart Study

**DOI:** 10.1371/journal.pone.0162845

**Published:** 2016-09-16

**Authors:** Arjun Gupta, Yehuda Herman, Colby Ayers, Muhammad S. Beg, Susan G. Lakoski, Shuaib M. Abdullah, David H. Johnson, Ian J. Neeland

**Affiliations:** 1 Department of Internal Medicine, University of Texas Southwestern Medical Center, Dallas, Texas, United States of America; 2 Collin College, Preston Road, Frisco, Texas, United States of America; 3 Department of Clinical Sciences, University of Texas Southwestern Medical Center, Dallas, Texas, United States of America; 4 Division of Oncology, University of Texas Southwestern Medical Center, Dallas, Texas, United States of America; 5 Department of Clinical Cancer Prevention & Cardiology, The University of Texas M.D. Anderson Cancer Center, Houston, Texas, United States of America; 6 Division of Cardiology, University of Texas Southwestern Medical Center, Dallas, Texas, United States of America; McMaster University, CANADA

## Abstract

**Purpose:**

Leptin dysregulation has been postulated to affect cancer risk through its effects on obesity and inflammation. Epidemiological data evaluating this relationship are conflicting and studies in non-white cohorts is lacking. Therefore, we examined the association of leptin with the risk of incident cancer in the multiethnic Dallas Heart Study (DHS).

**Methods:**

Participants enrolled in the DHS without prevalent cancer and with baseline leptin measurements were included. Incident cancer cases were identified through a systematic linkage of the DHS and the Texas Cancer Registry. Leptin was evaluated both as a continuous variable and in sex-specific quartiles. Multivariable Cox proportional hazards modeling was performed to examine the association between leptin levels with incident cancer after adjusting for age, sex, race, smoking status, alcohol use, family history of malignancy, body mass index (BMI), diabetes mellitus and C-reactive protein.

**Results:**

Among 2,919 participants (median age 44 years; 54% women; 70% nonwhite; median BMI 29.4 kg/m^2^), 190 (6.5%) developed cancer after median follow- up of 12 years. Median leptin levels were 12.9 (interquartile range [IQR] 5.8–29.5) ng/ml in the incident cancer group vs. 12.3 (IQR 5.4–26.4) ng/ml those without an incident cancer (p = 0.34). Leptin was not associated with cancer incidence in multivariable analysis (unit standard deviation increase in log-transformed leptin, hazard ratio 0.95; 95% confidence interval, 0.77–1.16; p = 0.60). No association was observed in analyses stratified by sex, race/ethnicity, diabetes, or obesity status.

**Conclusions:**

In this study of a predominantly minority population, no association between premorbid leptin levels and cancer incidence was demonstrated. Despite preclinical rationale and positive findings in other studies, this association may not replicate across all racial/ethnic populations.

## Introduction

Leptin, a polypeptide hormone predominantly secreted by white adipose tissue, is a key regulator of body weight homeostasis due to its effects on food intake and energy expenditure. Dysregulation in leptin metabolism has been linked to obesity, hyperinsulinemia and diabetes mellitus [1]. More recently its role in chronic inflammation, immunity, neoangiogenesis and tumorigenesis has been demonstrated [2]. The leptin receptor has been detected in both normal and malignant tissue [3]. Thus, it is hypothesized that leptin dysregulation may contribute to cancer risk. Obesity, as defined by body mass index (BMI) >30 kg/m^2^ is associated with increased cancer risk, and leptin may be a potential mediator of this association [3].

Studies evaluating the association of leptin and cancer have largely been retrospective and may be biased due to reverse causation due to the effect of cancer-associated weight loss on leptin levels [4]. Existing prospective studies reporting the association between leptin and cancer have lacked external validity due to relative lack of racial diversity in the cohorts. Results of these studies have been conflicting [5–13]. Only a single study conducted in Hong Kong evaluated pre-morbid leptin levels and risk of all-incident cancer and found no difference in leptin levels between individuals who developed cancer versus those who did not [14].

We aimed to prospectively study the relationship between pre-diagnostic plasma leptin levels and the risk of incident cancer among relatively young, multiethnic participants in the Dallas Heart Study (DHS).

## Materials and Methods

### Study Population

Details on the design of the DHS have been previously described [15]. Briefly, the DHS is a single site, multiethnic, population based probability sample of Dallas County residents (aged 18–65 years) with deliberate oversampling of non- Hispanic black participants.

The current study population was drawn from 3557 participants who completed DHS phase 1 (DHS-1) visits from 2000 to 2002, which included a computer-assisted survey, anthropometric and blood pressure measurements and laboratory testing. Participants without plasma leptin level assessment were excluded. Of the remaining participants, those with history of or present diagnosis of malignancy were also excluded. To account for cancers that may have been undetected at baseline, new cases of cancer diagnosed within 1 year after date of enrollment to DHS were excluded from the analysis (blanking period). After these exclusions, 2,919 participants were eligible for follow-up (Figure A in S1 File).

All participants provided written informed consent, and the University of Texas Southwestern Medical Center Institutional Review Board approved the protocol. All procedures performed in studies involving human participants were in accordance with the ethical standards of the institutional and/or national research committee and with the 1964 Helsinki declaration and its later amendments or comparable ethical standards.

Demographics, lifestyle and other risk factors were determined from a baseline questionnaire. Ethnicity was self-assigned in accordance with U.S. census categories. BMI was calculated as weight (kilograms) divided by the square of height (meters). Waist circumference (WC) and hip circumference (HC) were measured in centimeters and waist hip ratio (WHR) was calculated as ratio of WC/HC. Hypertension was defined as BP ≥140/90 mm Hg or taking antihypertensive medication(s). Diabetes mellitus was defined as a fasting serum glucose ≥126 mg/dl, self-reported diabetes, or taking hypoglycemic medication. Smoking was defined as cigarette use within the previous 30 days and/ or a lifetime history of having smoked ≥100 cigarettes. Alcohol use was determined in grams/week by self-report. Comorbid conditions were determined from self report, medication history and clinical assessment.

### Assessment of Leptin and other Biomarkers

Fasting blood samples were obtained from participants and collected in EDTA-containing tubes and stored at -80°C. A commercially available radioimmunoassay (Linco Research Inc., St. Charles, Missouri) was used to quantify total leptin levels according to manufacturer’s specification [16]. The lowest level of leptin that can be detected by this assay is 0.5 ng/mL and all values <0.5 ng/mL were designated 0.5 ng/mL (intra-assay and inter-assay coefficient of variation of 8.3% and 6.2%, respectively in men, and 3.4% and 4.6% in women). High sensitivity assay for C-reactive protein (CRP) was performed using the Roche/Hitachi 912 System (Roche Diagnostics, Indianapolis, Indiana) as described previously [16]. Samples were also analyzed for interleukin (IL)-6, adiponectin, and insulin levels [17,18].

### Cancer- outcomes

DHS was systematically linked to the Texas Cancer Registry (TCR) to determine cancer cases in the cohort [19]. The TCR is a population based registry of the State of Texas, and meets quality data standards of both the National Program of Cancer Registries (Center for Disease Control and Prevention) and North American Association of Central Cancer Registries (NAACCR). The Texas Cancer Incidence Reporting Act mandates health care facilities including hospitals, ambulatory surgical centers and cancer treatment centers to report to the TCR. All cancer cases identified by the TCR were classified as ‘prevalent’ or ‘incident’ based on date of cancer diagnosis in relation to date of enrollment to DHS. In cases with more than one known cancer, only the first cancer was included. Carcinoma in situ and skin cancers were not included. ‘*Obesity-associated cancers’* were defined as per the National Cancer Institute Obesity and Cancer Fact Sheet and included post-menopausal breast and endometrial cancers in women, along with esophageal, pancreatic, gall bladder, colorectal, kidney and thyroid cancer in men and women [20].

### Statistical Analysis

Baseline demographic, clinical, laboratory, and imaging variables between participants with and without incident cancer are expressed as median (25th, 75th percentile) or proportions and were compared using chi square tests or the Wilcoxon rank sum test, as appropriate. Univariate correlations were expressed using Pearson correlation coefficient for normally distributed parameters and Spearman correlation coefficient for non-normal distributed data as appropriate. Leptin levels were modeled following a natural logarithmic transformation. Because leptin levels are highly dependent on sex, sex-specific quartiles for the entire population were constructed. Kaplan Meier curves for the relation of sex-specific quartiles of leptin, to time to incident cancer cases were constructed and compared using the log-rank test. To better understand the relationship between leptin and BMI, we plotted the percentage of participants with a leptin level less than/greater than the sex-specific median by BMI categories (normal BMI 18.5–24.9 kg/m^2^, overweight 25–29.9 kg/m^2^, mild obesity 30–34.9 kg/m^2^, and severe obesity > = 35 kg/m^2^).Cox proportional hazards models were used to examine the unadjusted and multivariable adjusted associations between leptin and incident cancer and are reported as hazard ratios (HR) and 95% confidence intervals (CI). Leptin measures were analyzed both continuously per unit standard deviation (SD) increase in log-transformed variables, and as sex-specific quartiles. The primary outcome was any incident cancer. The secondary outcome was development of obesity-associated cancer. Cox proportional models were constructed such that the unadjusted model (model 1) was univariable in continuous analysis and sex-specific in quartile analysis. Models were sequentially adjusted for race (and sex in the case of continuous analysis) (model 2), age (model 3), family history of cancer; smoking and alcohol use (model 4), BMI (model 5), and diabetes mellitus and CRP levels (model 6). Formal testing for statistical interaction by sex, race (black/non-black), diabetes status, and obesity status was performed. Sensitivity analyses were performed by extending the blanking period to 2 years, including cancers diagnosed within the 1 year blanking period, excluding lung and esophageal cancers (associated with lower BMI), and hematological cancers from the analysis, excluding breast and prostate cancers (associated with screening procedures) from the analysis, and analyzing non-smokers only. Since the association between leptin and cancer may be mediated via obesity or inflammation, we also analyzed the relation of BMI, waist circumference, waist-hip ratio, CRP levels and interleukin-6 levels with incident cancer. P-values of <0.05 were considered statistically significant. All analyses were performed using SAS version 9.2 (SAS Corporation, Cary, North Carolina).

## Results and Discussion

The study cohort consisted of 2,919 cancer-free individuals at inception, median age of 41 years (IQR 35, 49). The cohort was 51% Black, 30% White and 17% Hispanic and 54% were female. The mean body mass index was 29.4 kg/m^2^ (range 14.5–65.2 kg/m^2^). Characteristics of the cohort stratified by development of cancer are presented in Table 1. One hundred and ninety individuals (6.5%) developed an incident cancer in the median follow-up period of 12.0 years [IQR 11.6, 12.5] after a 1-year blanking period, of which 55% were female and 69% were non-white. The median time to cancer diagnosis was 8.1 years (IQR 4.5, 11.7). Of the 190 patients who developed cancer, 79 (42%) were ‘obesity- associated’ cancers. The most common primary cancer sites in women and men were breast and prostate (24% and 18% of total cancers), respectively. Further details regarding the primary site of cancer are presented in Table 2. These data are similar to the general distribution of cancers seen in the overall TCR registry. Those who developed cancer were more likely to be older, smoke, have a family history of cancer and higher prevalence of diabetes mellitus, hypertension and hyperlipidemia compared with those that did not develop cancer. They were also more likely to have higher WC and WHR; no significant differences in serum biomarker levels were observed (Table 1).

**Table 1:** Baseline characteristics of participants with and without incident cancer in the DHS (data are reported as median (interquartile range) or number (%), as appropriate).

Characteristic	No cancer (n = 2,729)	Incident cancer (n = 190)	P-value
**Clinical characteristics**			
Age (years)	43 (36, 51)	52 (45, 57)	<0.01
Male	1248 (45.7%)	85 (44.7%)	0.82
Race			
Black	1372 (50.3%)	108 (56.9%)	0.08
White	825 (30.2%)	59 (31.1%)	0.81
Hispanic	477 (17.5%)	17 (9%)	<0.01
Other	55 (2.0%)	6 (3.2%)	0.29
Smoking	755 (27.7%)	65 (34.6%)	0.05
Alcohol use	1880 (69.0%)	131 (69.3%)	1.00
Diabetes mellitus	306 (11.2%)	38 (20%)	<0.01
Hypertension	885 (32.9%)	101 (54%)	<0.01
Hyperlipidemia	341 (12.5%)	47 (24.7%)	<0.01
Physical activity (MET-min/wk)	133 (0, 585)	113 (0, 480)	0.43
Family history of cancer	585 (21.4%)	62 (32.6%)	<0.01
**Biochemical characteristics**			
High-sensitivity C-reactive protein (mg/dL)	2.7 (1.2, 6.7)	3.6 (1.4, 8.3)	0.71
Interleukin-6 (pg/mL)	16.7 (0.0–34.9)	19.9 (0, 41.5)	0.82
Adiponectin (ug/mL)	6.4 (4.4, 9.5)	6.4 (4.5, 9.4)	0.73
Leptin (ng/mL)	12.3 (5.4, 26.4)	12.9 (5.8, 29.5)	0.34
Insulin (uIU/mL)	12.6 (7.4, 20.6)	14.2 (8.1, 23.5)	0.06
**Measures of adiposity**			
Body weight (kg)	83.2 (70.8, 98.4)	82.7 (71.2, 100.4)	0.41
Body mass index (kg/ m^2^)	29.4 (25.4, 34.6)	30.0 (25.2, 35.6)	0.34
Waist circumference (cm)	98 (87.5, 110)	100.8 (90, 113)	0.02
Waist hip ratio	0.9 (0.9, 1.0)	0.9 (0.9, 1.0)	<0.01

**Table 2:** Distribution of incident cancers by primary site, with sex-specific leptin levels^1^.

Type of cancer	Anatomical sites included	Number (% of total) of incident cancers	Median Leptin (IQR) (ng/mL), females	Median Leptin (IQR) (ng/mL), males
Breast	Breast	45 (24)	26.8 (10.2–45.8)	-
Prostate	Prostate	35 (18)	-	6.0 (3.1–10.8)
Lung	Lung	16 (8)	33.1 (15.3–37.3)	2.5 (1.1–8.4)
Genitourinary	Gynecological, kidney, urinary bladder	28 (15)	29.5 (14.4–42.2)	5.0 (2.3–11.2)
Gastrointestinal	Esophagus, stomach, small intestine, colon, rectum, anus, liver, pancreas, gallbladder	31 (16)	21.0 (11.5–25.4)	5.8 (2.7–15.3)
Hematological	Leukemia, Lymphoma (Hodgkin and non- Hodgkin)	16 (8)	34.4 (18.9–50.3)	4.5 (0.5–9.8)
Others	Brain, thyroid, head and neck, not otherwise specified	19 (10)	37.6 (19.6–42.2)	4.5 (2.0–7.0)

^1^All breast cancer cases were post-menopausal

### Relation with Leptin levels

Median leptin levels were 12.9 (IQR 5.8, 29.5) ng/ml in the cancer group and 12.3 (IQR 5.4, 26.4) ng/ml in non-cancer group (p = 0.34). Median leptin levels were higher in females (females, 23.2 [IQR 13.7–37.2]; males 5.6 [IQR 2.7–9.9]; p<0.01) and blacks (blacks, 15.7 [IQR 6.2–32.2] whites, 10.3 [IQR 5.0–20.9]; Hispanics, 11.1 [5.4–22.8]; p<0.01). Characteristics of the cohort across sex-specific quartiles of leptin are given in Table A in S1 File. In individuals that developed cancer, women had higher leptin levels than men for each primary cancer site (Table 2). All breast cancers were in post-menopausal women.

In both men and women, there was a significant trend towards higher percentage of individuals with a leptin level greater than the sex-specific median as BMI category increased from 8.2% of women and 12.9% of men among those with BMI 18.5–24.9 kg/m^2^ to 89.7% of women and 97.3% of men among those with BMI >35 kg/m^2^ (p < 0.0001) (Figure B in S1 File).

**Fig 1:**
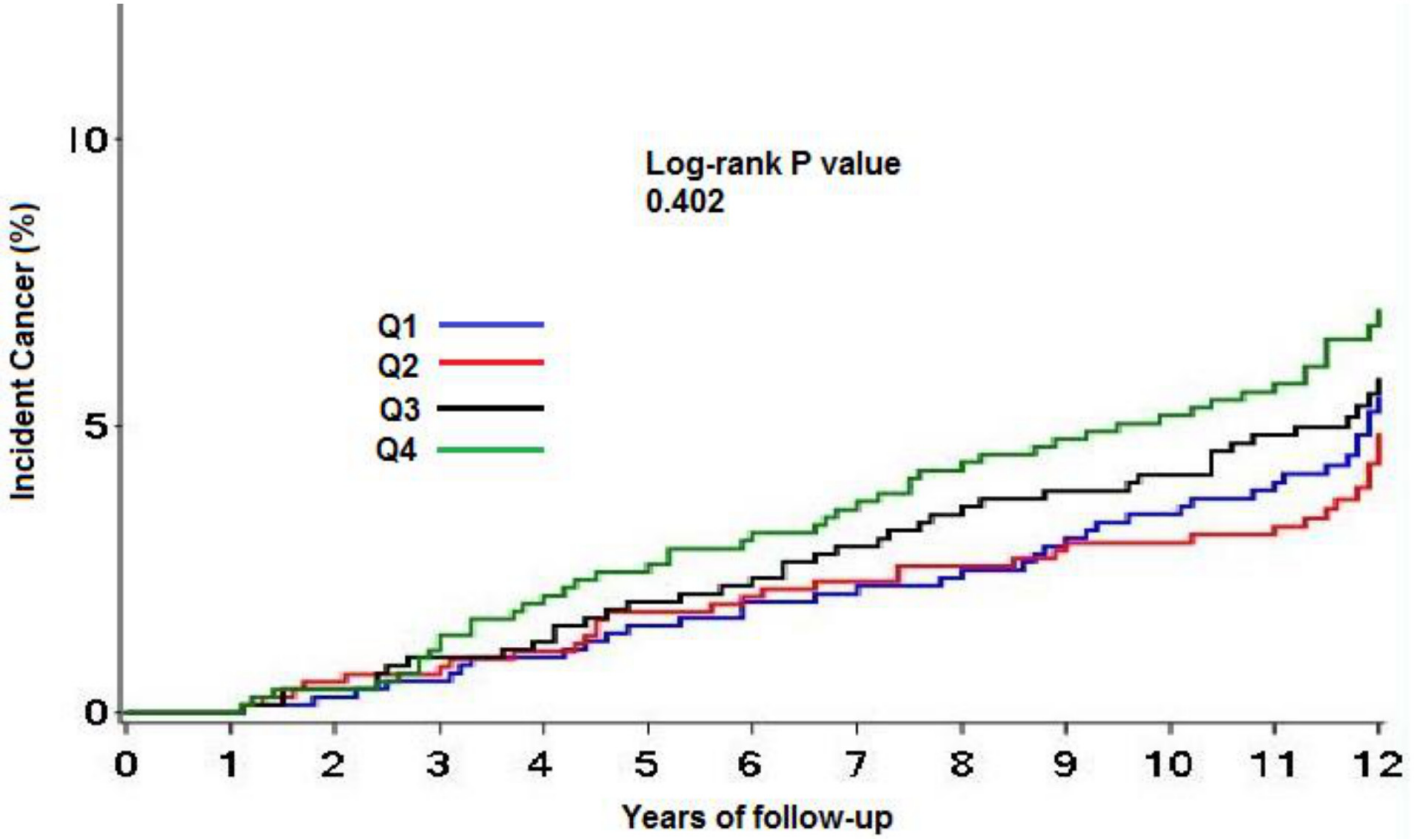
Kaplan Meier curve of time to cancer by sex- specific leptin quartiles. Vertical axes show the percentage of subjects developing cancer in each quartile, horizontal axes represent years of follow- up. Median interval to cancer diagnosis was 8.1 years. (Q1 refers to quartile 1, Q2 to quartile 2, etc.).

**Fig 2:**
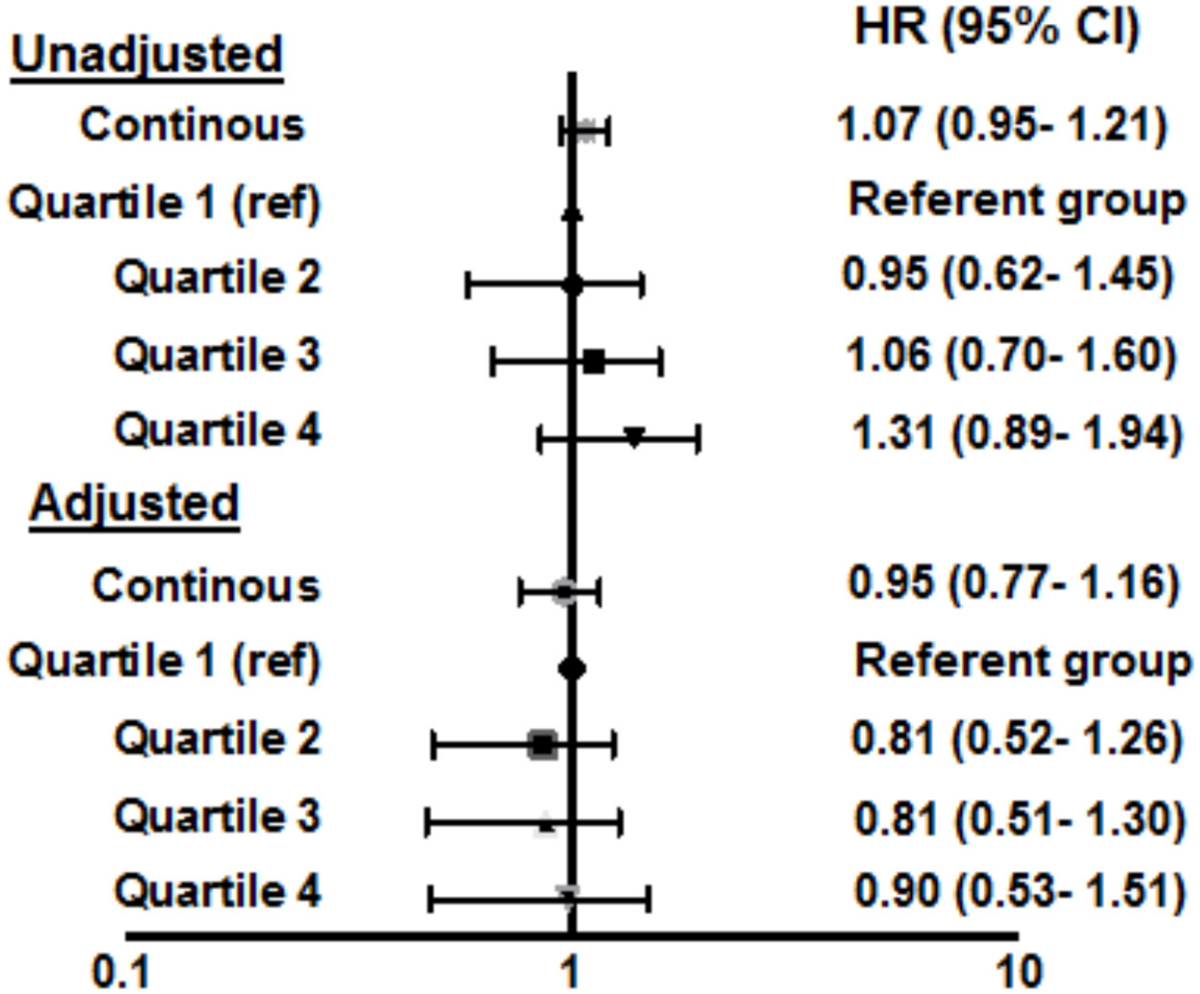
Forrest plot of the risk for incident cancer per 1-unit standard deviation increase in log-transformed leptin levels, and by quartiles, before and after adjustment for clinical and demographic variables. Model is adjusted for sex, race, age, smoking status, alcohol use, family history of cancer, body mass index, diabetes mellitus and C-reactive protein. Data is given as hazard ratio (95% confidence intervals).

Fig 1 shows the Kaplan Meier curves for incident cancer by sex-specific quartiles of leptin; the risk of incident cancer were 6.2% for Q1, 5.7% for Q2, 6.4% for Q3, and 7.8% for Q4 (log-rank p = 0.40). In the overall population, there was no univariate association between unit SD difference in log-transformed leptin and incident cancer, HR 1.07 (95% CI, 0.95–1.21, p = 0.27), and similar results were obtained in fully-adjusted models (HR 0.95, 95% CI 0.77, 1.16, p = 0.60) (Fig 2, and Table B in S1 File). On review of the models, it appeared BMI attenuated the relationship between leptin and incident cancer the most. No association between unit SD increase in log-transformed leptin and obesity- associated cancers was seen (fully adjusted model, HR 1.00, 95% CI 0.69–1.46, p = 0.99) (Table C in S1 File). In analysis stratified by sex (male vs female), race (black vs non-black), diabetes status (present vs absent) or BMI (normal weight vs. overweight/obese) (Table D in S1 File), formal testing did not reveal significant interaction between leptin and any of these variables; however, there was a non-significant trend towards a positive association between leptin and cancer in females, blacks and diabetics. Sensitivity analysis performed including cancers diagnosed within 1 year of study enrollment, extending the blanking period to 2 years, excluding lung, esophageal and hematological cancers, and excluding breast and prostate cancers, showed generally similar results (Table E in S1 File). No significant association was noted between BMI, WC, WHR, CRP, IL-6 levels, and incident cancer (Table F in S1 File). Findings remained largely unchanged when analysis was restricted to non-smokers only (Table G in S1 File).

In this prospective multiethnic cohort study, plasma leptin levels were not associated with development of incident cancer over a follow up of 12 years. We also did not observe an association between leptin and future development of obesity-associated cancers, although the number of individual cancers was relatively small. Our findings were similar across sex and racial/ethnic groups.

Preclinical studies have linked leptin to pro-neoplastic properties, including neoangiogenesis and vascular remodeling and have described its behavior as a mitogen and transforming factor. In vitro studies using multiple cancer cell lines have identified cell proliferation and invasion in cells exposed to leptin [2]. A pivotal study found that leptin led to increased DNA synthesis and cell growth in colon cancer cell lines in-vitro, but did not promote tumorigenesis in xenograft models [21]. In line with the above study, the reported association between leptin and cancer in human population based studies has been inconsistent.

Several studies have shown a null relationship between leptin levels and prostate cancer [4,22,23]. This was confirmed in a recent large case-control study from the Health Professionals Follow- up Study [24]. Stattin, et al. have demonstrated a potentially non-linear relationship between leptin levels and prostate cancer, with very elevated leptin levels associated with reduced cancer incidence. However, elevated estrogen levels associated with obesity and higher leptin levels may contribute to breast/ uterine carcinogenesis, and account for a more linear relationship in women as demonstrated by the positive association of leptin with endometrial and breast cancer on meta-analysis [25,26], These data are limited, however, due to significant heterogeneity in findings, evidence of publication bias and incomplete adjustment for BMI. Wulaningsih, et al. have shown that leptin was a predictor of cancer-related mortality in women, but not men in the Third National Health and Examination Survey [27]. Given the known sex variation in leptin levels between men and women, it is reasonable to consider that leptin could have a positive association in women where leptin levels are higher, but not in men. Both breast and endometrial cancer are also obesity-associated cancers raising the possibility that leptin increased the risk of obesity-associated cancer but not all cancers. However, a recent systematic review and meta-analysis of 13 studies did not find an association between leptin levels and risk of colorectal cancer, a classic obesity- associated cancer [28]. We also did not see an association between leptin and cancer in either sex, or on analyzing obesity-associated cancers alone. There was a trend toward a higher point estimate for hazard in quartile 1 of leptin (versus quartiles 2,3 and 4) in obesity-associated cancers and this may represent a population with an excess of risk factors for incident cancer, such as smoking, alcohol use and other unmeasured risk factors. This may partially explain this reverse J shaped relationship, and needs further study. Other studies have found that even cancers associated with lower BMI (e.g. lung cancer) have been associated with increased serum and tissue leptin levels [29]. However, our results were insensitive to inclusion or exclusion of cancers associated with lower BMI. Although stratified analysis did not show a significant interaction between sex/ diabetes status and leptin, females and individuals with diabetes had non-significant trends towards association of leptin and cancer. Leptin levels are known to be higher in women/ diabetics and it is plausible that a threshold level of leptin may exist after which it becomes more associated with cancer.

Although leptin tracks closely with BMI and most individuals with high leptin have elevated BMI (~8% of women and 13% of men in our study), the results of our study were not likely altered by collinearity between BMI and leptin since neither BMI nor leptin (after BMI adjustment) were associated with incident cancer. The ‘obesity paradox’ refers to a finding that obesity is associated with improved outcomes in certain chronic diseases such as congestive heart failure, chronic kidney disease and chronic obstructive pulmonary disease, although there is concern that it is largely a statistical flaw due to confounding (especially smoking and lower BMI in the years before death). In our study, quartile 1 of leptin had a disproportionately higher percentage of smokers and alcohol users, and the lowest BMI. Thus, the U shaped curves may be explained by higher smoking and alcohol use, and lower BMI/frailty in the quartile 1 group, which may be a reflection of the “obesity paradox”. However, we attempted to address this by adjusting for these factors, and indeed in Model 5 of Table B in S1 File, we saw that the U shape was largely abolished. Since leptin levels correlate with BMI, the U shaped in incidence could track with the obesity paradox although clearly even after maximal adjustment, the U shape is slightly present. Further studies are needed to specifically address this question in leptin (versus obesity).

Most of the prior studies have lacked racial diversity- the Health Professionals Follow- up Study comprised >90% whites, while the Third National Health and Examination Survey included 76% whites- thus limiting the external validity of these studies to populations with larger non-white representation, such as the DHS. Cancer cachexia is associated with altered leptin metabolism and variation in plasma leptin levels [30,31]. We aimed to avoid this confounding by subclinical disease in our study by eliminating cancer cases diagnosed within 1 year of study enrolment from the analysis. Studies have had varying censoring periods and some have measured exposure at cancer diagnosis. There may be an age-dependant contribution of leptin to carcinogenesis; Bologna et al demonstrated that serum leptin values were better associated with prostate cancer risk in older patients, compared to younger patients [32]. The younger age of our cohort compared to other cohorts may contribute to our observed lack of association. Differences in techniques of measuring leptin, and local leptin concentrations, leptin receptor status and downstream signaling may also explain the heterogeneity in study findings. We used a well validated radioimmunoassay to measure leptin level in our study [33]. Serum leptin receptor levels may be useful to predict cancer risk but the data for this is limited [34]. Polymorphisms in genes coding for leptin and the leptin receptor have been associated with cancer incidence [35,36] and mortality [37]. We were unable to evaluate the significance of serum leptin receptor levels and genetic polymorphisms as the DHS did not record these. Although the lack of an observed association between leptin and cancer in our study could be due to type II error, a false negative result is unlikely as our study had sufficient power (80%) to detect a 20% relative difference in cancer risk.

Strengths of the study include a large, multiethnic population cohort with accurate leptin measurement with close follow-up for the development of cancer. The intentional oversampling of the black population in the DHS cohort provides a unique opportunity to evaluate this relationship in less well represented racial/ethnic groups.

Limitations include the observational design which precludes ability to ascribe causation. DHS participants who were diagnosed with cancer outside the State of Texas may not have been captured so a degree of ascertainment bias may be present. A cut off period of 1 year between enrollment in the DHS and date of cancer diagnosis was used to separate ‘incident’ from ‘prevalent’ cancer cases; this minimized the risk of underlying malignancy from confounding leptin levels, but reduced the total number of incident cancers and our ability to detect a statistically significant difference. However, it is unlikely that inclusion of cancers diagnosed within one year of leptin measurement would materially alter the results. Furthermore, the relatively young age of our cohort is likely responsible for a lower cancer incidence rate than some other databases, which also precluded our ability to perform individual analyses for each cancer site. However it is unlikely that this dataset did not have a wide enough range of BMI to observe an association with leptin. Finally, since our study did not include South or East Asians, we are unable to determine the impact of leptin on cancer in these racial/ethnic groups.

## Conclusions

Our study in a large multiethnic cohort did not observe an association between plasma leptin level and future cancer risk. Further studies are necessary to understand the mechanism of carcinogenesis in obesity and insulin resistance in a prospective manner.

## Supporting Information

S1 File Supplementary Tables and Figures. (DOCX)
