# Supplementary material for: Plasma Leptin Levels and Risk of Incident Cancer: Results from the Dallas Heart Study
Source: PLoS One. 2016 Sep 16;11(9):e0162845. doi: 10.1371/journal.pone.0162845 (PMC5026337; doi:10.1371/journal.pone.0162845)
Supplement: S1 File — (DOCX) [file pone.0162845.s001.docx]

**S1 File: Supplementary data**

Supplementary Table A: Demographic, clinical, imaging and biochemical characteristics of study population at baseline exam stratified by sex- specific quartiles of leptin (data are reported as median (interquartile range) or number (%), as appropriate)

Supplementary Table B: Cox proportional hazard models of leptin level and incident cancer

Supplementary Table C: Cox proportional hazard models of leptin level and incident obesity-associated cancer

Supplementary Table D: Fully adjusted cox proportional hazard models of leptin level and incident cancer, stratified by sex, race, diabetes mellitus and body mass index

Supplementary Table E: Sensitivity analysis for fully adjusted cox proportional hazard models of leptin level and incident cancer

Supplementary Table F: Continuous cox proportional hazard models of body mass index, waist circumference, waist hip ratio, C-reactive protein and interleukin-6 with incident cancer

Supplementary Table G: Cox proportional hazard models of leptin level and incident cancer in non-smokers

Supplementary Figure A: Flow Diagram of the Dallas Heart Study Cohort

Supplementary Figure B: Percentage of men and women in each body mass index category with leptin levels greater than the sex-specific median levels

| **Characteristic** | **Overall**  **(n=2919)** | **Quartile 1 (n=722)** | **Quartile 2 (n=741)** | **Quartile 3 (n=723)** | **Quartile 4 (n=733)** | **P-value of trend** |
| --- | --- | --- | --- | --- | --- | --- |
| **Leptin characteristics** | | | | | | |
| Leptin range - Overall (ng/mL) |  | 3.1 (1.5, 9.0) | 14.5 (4.1, 18.6) | 24.1 (7.7, 30.0) | 39.3 (15.7, 49.9) | <0.01 |
| Leptin range - Females (ng/mL) |  | 0.5- 13.7 | 13.8- 23.1 | 23.2- 37.1 | 37.2- 156.5 | <0.01 |
| Leptin range – Males (ng/mL) |  | 0.5- 2.6 | 2.7- 5.6 | 5.7- 9.8 | 9.9- 119.6 | <0.01 |
| **Clinical characteristics** | | | | | | |
| Age (years) | 44 (37, 52) | 41 (35, 49) | 44 (37- 52) | 44 (37- 52) | 46 (38-53) | <0.01 |
| Male | 1333 (45.7%) | 325 (45.0%) | 346 (46.7%) | 327 (45.2%) | 335 (45.7%) | 0.95 |
| Race |  |  |  |  |  |  |
| Black | 1480 (50.7%) | 328 (45.4%) | 330 (44.5%) | 366 (50.6%) | 456 (62.2%) | <0.01 |
| White | 884 (30.2%) | 240 (33.2%) | 245 (33.1%) | 217 (30.0%) | 182 (24.8%) | <0.01 |
| Hispanic | 494 (16.9%) | 135 (18.7%) | 149 (20.1%) | 127 (17.6%) | 83 (11.3%) | <0.01 |
| Other | 61 (2.1%) | 19 (2.6%) | 17 (2.3%) | 13 (1.8%) | 12 (1.6%) | <0.14 |
| Smoking | 820 (28.1%) | 288 (40.0%) | 215 (29.1%) | 160 (22.2%) | 157 (21.4%) | <0.01 |
| Alcohol use | 2011 (68.9%) | 540 (74.9%) | 516 (69.8%) | 480 (66.7%) | 475 (64.9%) | <0.01 |
| Diabetes mellitus | 344 (11.8%) | 43 (6.0%) | 83 (11.2%) | 96 (13.3%) | 122 (16.7%) | <0.01 |
| Hypertension | 986 (33.8%) | 141 (19.7%) | 218 (30.0%) | 285 (39.8%) | 342 (47.6%) | <0.01 |
| Hyperlipidemia | 388 (13.3%) | 55 (7.6%) | 106 (14.3%) | 105 (14.5%) | 122 (16.7%) | <0.01 |
| Physical activity  (MET-min/wk) | 133 (0, 572) | 240 (0, 780) | 105 (0, 585) | 120 (0, 540) | 88 (0, 479) | <0.01 |
| Family history of cancer | 647 (22.2%) | 139 (19.3%) | 173 (23.4%) | 169 (23.4%) | 166 (22.7%) | 0.14 |
| **Biochemical characteristics** | | | | | | |
| High-sensitivity C-reactive protein (mg/dL) | 2.8 (1.2, 6.7) | 1.4 (0.6, 3.5) | 2.4 (1.0, 4.8) | 3.2 (1.6, 7.4) | 5.6 (2.4, 11.9) | <0.01 |
| Interleukin-6 (pg/mL) | 16.9 (0.0-35.4) | 17.0 (0, 37.4) | 15.5 (0, 33.0) | 17.8 (0, 36.4) | 16.6 (0, 35.1) | 0.95 |
| Adiponectin (ug/mL | 6.4 (4.4- 9.5) | 8.2 (5.5, 12.5) | 6.4 (4.4, 9.6) | 6.0 (4.0, 8.5) | 5.5 (4.0, 8.1) | <0.01 |
| Insulin (uIU/mL) | 12.7 (7.5, 20.8) | 6.5 (4.2, 9.6) | 10.6 (7.2, 16.7) | 15.2 (10.2,21.5) | 21.4 (14.9, 29.7) | <0.01 |
| **Measures of adiposity** | | | | | | |
| Body weight (kg) | 83.0 (70.8- 98.8) | 66.7 (58.5, 75.3) | 78.2 (69.9, 87.0) | 88.9 (78.9, 101.3) | 104.2 (92.4, 119.9) | <0.01 |
| Body mass index ( kg/ m^2^) | 29.4(25.4, 34.7) | 23.8 (21.6, 26.3) | 27.8 (25.3, 30.5) | 31.2 (28.5, 35.2) | 36.6 (32.7, 42.4) | <0.01 |
| Waist circumference (cm) | 98 (88, 110) | 83.5 (76.5, 90.5) | 94 (88, 101) | 102.5 (95, 111.5) | 114(105, 123.5) | <0.01 |
| Waist hip ratio | 0.91 (0.85- 0.96) | 0.87 (0.81- 0.92 | 0.91 (0.86, 0.96) | 0.92 (0.86, 0.97) | 0.93 (0.87, 0.99) | <0.01 |

**Supplementary Table A:** Demographic, clinical, imaging and biochemical characteristics of study population at baseline exam stratified by sex- specific quartiles of leptin (data are reported as median (interquartile range) or number (%), as appropriate)

**Supplementary Table B:** Cox proportional hazard models of leptin level and incident cancer

| **Leptin measure** | **Model 1**  **HR (95% CI)** | **Model 2**  **HR (95% CI)** | **Model 3**  **HR (95% CI)** | **Model 4**  **HR (95% CI)** | **Model 5**  **HR (95% CI)** | **Model 6**  **HR (95% CI)** |
| --- | --- | --- | --- | --- | --- | --- |
| **Continuous (per log increase)** | 1.07  (0.95- 1.21) | 1.09  (0.93- 1.27) | 0.99  (0.85- 1.16) | 1.04  (0.89- 1.22) | 0.95  (0.77- 1.16) | 0.95  (0.77- 1.16) |
| **Quartile 2 (vs quartile 1)** | 0.95  (0.62- 1.45) | 0.96  (0.63- 1.46) | 0.81  (0.53- 1.23) | 0.86  (0.56- 1.32) | 0.81  (0.52- 1.25) | 0.81  (0.52- 1.26) |
| **Quartile 3 (vs quartile 1)** | 1.06  (0.70- 1.60) | 1.05  (0.70- 1.58) | 0.89  (0.58- 1.34) | 0.94  (0.61- 1.44) | 0.83  (0.52- 1.33) | 0.81  (0.51- 1.30) |
| **Quartile 4 (vs quartile 1)** | 1.31  (0.89- 1.94) | 1.27  (0.86- 1.88) | 1.00  (0.67- 1.49) | 1.11  (0.74- 1.66) | 0.90  (0.53- 1.51) | 0.90  (0.53- 1.51) |

Data include both per 1-standard deviation increase in log-transformed leptin and sex-specific quartile analysis; HR (95% CI) - hazard ratio (95% confidence interval)

Model 1- unadjusted (univariable in continuous and sex-specific in quartile analysis)

Model 2- additionally adjusted for race (and sex in continuous analysis)

Model 3- additionally adjusted for age

Model 4- additionally adjusted for smoking, alcohol and family history of cancer

Model 5- additionally adjusted for body mass index,

Model 6- additionally adjusted for diabetes mellitus and C-reactive protein level

(chi-square value for BMI 4.0, CRP 0.2 and DM 1.3) in fully-adjusted models.

**Supplementary Table C:** Cox proportional hazard models of leptin level and obesity-associated cancer

| **Leptin measure** | **Model 1**  **HR (95% CI)** | **Model 2**  **HR (95% CI)** | **Model 3**  **HR (95% CI)** | **Model 4**  **HR (95% CI)** | **Model 5**  **HR (95% CI)** |
| --- | --- | --- | --- | --- | --- |
| **Continuous (per log increase)** | 1.46  (1.17- 1.82) | 1.12  (0.86- 1.48) | 1.04  (0.79- 1.37) | 1.12  (0.84- 1.49) | 1.00  (0.69- 1.46) |
| **Quartile 2 (vs quartile 1)** | 0.71  (0.37- 1.35) | 0.71  (0.37- 1.35) | 0.61  (0.32- 1.16) | 0.64  (0.33- 1.24) | 0.53  (0.27- 1.05) |
| **Quartile 3 (vs quartile 1)** | 0.81  (0.44- 1.52) | 0.82  (0.44- 1.52) | 0.71  (0.38- 1.33) | 0.77  (0.40- 1.45) | 0.50  (0.24- 1.02) |
| **Quartile 4 (vs quartile 1)** | 1.04  (0.58- 1.87) | 1.06  (0.59- 1.90) | 0.87  (0.48- 1.58) | 0.93  (0.51- 1.72) | 0.50  (0.22- 1.11) |

Data include both per 1-standard deviation increase in log-transformed leptin and sex-specific quartile analysis; HR (95% CI) - hazard ratio (95% confidence interval)

Model 1- unadjusted (univariable in continuous and sex-specific in quartile analysis)

Model 2- additionally adjusted for race (and sex in continuous analysis)

Model 3- additionally adjusted for age

Model 4- additionally adjusted for smoking, alcohol and family history of cancer

Model 5- additionally adjusted for body mass index, diabetes mellitus and C-reactive protein level

**Supplementary Table D:** Fully adjusted cox proportional hazard models of leptin level and incident cancer, stratified by sex, race, diabetes status and body mass index

|  | **HR (95% CI)** | | | | | | | |
| --- | --- | --- | --- | --- | --- | --- | --- | --- |
|  | **Sex** | | **Race** | | **Diabetes Mellitus** | | **Body mass index** | |
| **Leptin measure** | **Male** | **Female** | **Black** | **Non-black** | **Yes** | **No** | **<25 kg/m^2^** | **>25 kg/m^2^** |
| **Number of incident cancers/ total** | **85/ 1333** | **105/ 1586** | **108/1480** | **82/1439** | **38/344** | **152/2574** | **51/ 780** | **139/ 2118** |
| **Continuous (per unit log increase)** | 0.94  (0.72- 1.23) | 1.11  (0.76- 1.62) | 1.05  (0.80- 1.37) | 0.84  (0.60- 1.18) | 1.51  (0.87- 2.62) | 0.88  (0.71- 1.11) | 1.04  (0.73- 1.49) | 0.96  (0.72- 1.28) |
| **Quartile 2 (vs quartile 1)** | 0.91  (0.49- 1.71) | 0.79  (0.42 1.47) | 1.40  (0.77- 2.52) | 0.42  (0.21- 0.83) | 1.35  (0.49- 3.74) | 0.85  (0.53- 1.37) | 1.59  (0.69- 3.65) | 0.79  (0.48- 1.32) |
| **Quartile 3 (vs quartile 1)** | 0.78  (0.38- 1.57) | 0.89  (0.46- 1.71) | 0.83  (0.42- 1.65) | 0.65  (0.33- 1.25) | 1.37  (0.48- 3.96) | 0.70  (0.41- 1.20) | 1.15  (0.46- 2.84) | 0.83  (0.50- 1.39) |
| **Quartile 4 (vs quartile 1)** | 0.85  (0.39- 1.87) | 0.94  (0.54- 2.44) | 1.46  (0.72- 2.95) | 0.56  (0.26- 1.23) | 1.98  (0.64- 6.13) | 0.75  (0.42- 1.36) | 1.05  (0.40- 2.72) | 0.88  (0.51- 1.53) |
| **Interaction p-value** | 0.30 | | 0.68 | | 0.21 | | 0.23 | |

Data include both per 1-standard deviation increase in log-transformed leptin and sex-specific quartile analysis; HR (95% CI) - hazard ratio (95% confidence interval)

Models adjusted for sex, race, age, smoking, alcohol, family history of cancer, body mass index, diabetes mellitus and C-reactive protein level (excluding the variable being stratified)

**Supplementary Table E:** Sensitivity analysis for fully adjusted cox proportional hazard models of leptin level and incident cancer

|  | **HR (95% CI)** | | | |
| --- | --- | --- | --- | --- |
| **Leptin measure** | **Including cancers diagnosed within 2 years of enrollment** | **Including cancer diagnosed within 1 year of enrollment** | **Excluding lung, esophageal and hematological cancer** | **Excluding breast and prostate cancer** |
| **Continuous (per unit log increase)** | 0.95  (0.77- 1.11) | 0.92  (0.73- 1.14) | 0.94  (0.75- 1.16) | 0.86  (0.66- 1.12) |
| **Quartile 2 (vs quartile 1)** | 0.76  (0.51- 1.14) | 0.75  (0.49- 1.19) | 0.74  (0.47- 1.17) | 0.84  (0.48- 1.47) |
| **Quartile 3 (vs quartile 1)** | 0.80  (0.45- 1.30) | 0.77  (0.47- 1.26) | 0.81  (0.50- 1.31) | 0.69  (0.37- 1.28) |
| **Quartile 4 (vs quartile 1)** | 0.90  (0.53- 1.43) | 0.86  (0.50- 1.48) | 0.85  (0.49- 1.50) | 0.78  (0.40- 1.54) |

Data include both per 1-standard deviation increase in log-transformed leptin and sex-specific quartile analysis; HR (95% CI) - hazard ratio (95% confidence interval)

Models adjusted for sex, race, age, smoking, alcohol, family history of cancer, body mass index, diabetes mellitus and C-reactive protein level

**Supplementary Table F:** Continuous cox proportional hazard models of body mass index, waist circumference, waist hip ratio, C-reactive protein and interleukin-6 with incident cancer

|  | **Body mass index** | **Waist circumference** | **Waist hip ratio** | **C-reactive protein** | **Interleukin-6** |
| --- | --- | --- | --- | --- | --- |
| **Unadjusted model** | 1.03  (0.99- 1.06) | 1.01  (1.00- 1.02) | 0.98  (0.79- 1.21) | 1.03  (0.99- 1.07) | 0.92  (0.73- 1.15) |
| **Maximally adjusted model** | 1.01  (0.98- 1.05) | 1.38  (0.89- 2.13) | 1.28  (0.98- 1.69) | 0.99  (0.95- 1.04) | 0.96  (0.84- 1.09) |

Data for 1-standard deviation increase in body mass index, waist circumference, waist hip ratio, and for log-transformed C-reactive protein and interleukin-6; data reported as hazard ratio (95% confidence interval)

Fully adjusted models include age, race, sex, smoking, alcohol, smoking, family history of cancer, body mass index, diabetes mellitus and C-reactive protein level (adjustment for body mass index and C-reactive protein not done in their respective models)

**Supplementary Table G:** Cox proportional hazard models of leptin level and incident cancer in non-smokers

| **Leptin measure** | **Model 1**  **HR (95% CI)** | **Model 2**  **HR (95% CI)** | **Model 3**  **HR (95% CI)** | **Model 4**  **HR (95% CI)** | **Model 5**  **HR (95% CI)** |
| --- | --- | --- | --- | --- | --- |
| **Continuous (per log increase)** | 1.16  (0.95- 1.32) | 1.27  (1.03- 1.59) | 1.15  (0.93- 1.43) | 1.16  (0.93- 1.44) | 1.13  (0.86- 1.49) |
| **Quartile 2 (vs quartile 1)** | 0.96  (0.56- 1.66) | 0.96  (0.56- 1.66) | 0.81  (0.47- 1.40) | 0.82  (0.47- 1.41) | 0.81  (0.46- 1.42) |
| **Quartile 3 (vs quartile 1)** | 1.11  (0.66- 1.87) | 1.10  (0.66- 1.87) | 0.93  (0.55- 1.58) | 0.94  (0.60- 1.59) | 0.89  (0.50- 1.58) |
| **Quartile 4 (vs quartile 1)** | 1.60  (0.98- 2.59) | 1.58  (0.97- 2.59) | 1.22  (0.74- 2.00) | 1.23  (0.75- 2.03) | 1.19  (0.63- 2.25) |

Data include both per 1-standard deviation increase in log-transformed leptin and sex-specific quartile analysis; HR (95% CI) - hazard ratio (95% confidence interval)

Model 1- unadjusted (univariable in continuous and sex-specific in quartile analysis)

Model 2- additionally adjusted for race (and sex in continuous analysis)

Model 3- additionally adjusted for age

Model 4- additionally adjusted for alcohol and family history of cancer

Model 5- additionally adjusted for body mass index, diabetes mellitus and C-reactive protein level


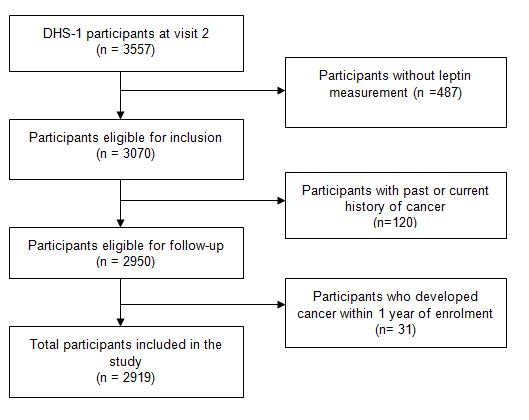


**Supplementary Figure A**: Flow Diagram of the Dallas Heart Study Study Cohort


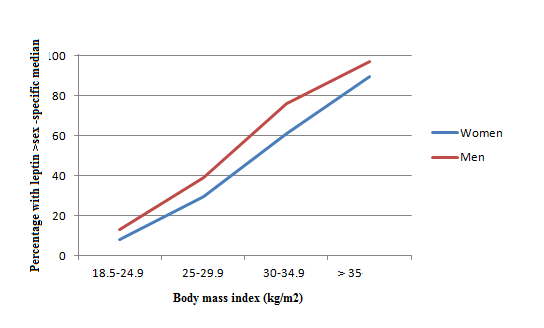


**Supplementary Figure B**: Percentage of men and women in each body mass index category with leptin levels greater than the sex-specific median level
